# Supplementary material for: Amphetamine disrupts dopamine axon growth in adolescence by a sex-specific mechanism in mice
Source: Nat Commun. 2023 Jul 7;14:4035. doi: 10.1038/s41467-023-39665-1 (PMC10329029; doi:10.1038/s41467-023-39665-1)
Supplement: Supplementary file 1 — Supplementary Information [file 41467_2023_39665_MOESM1_ESM.pdf]

Amphetamine disrupts dopamine axon growth in adolescence by a sex-specific mechanism in mice

Supplementary Information

Supplementary Figures and Legends

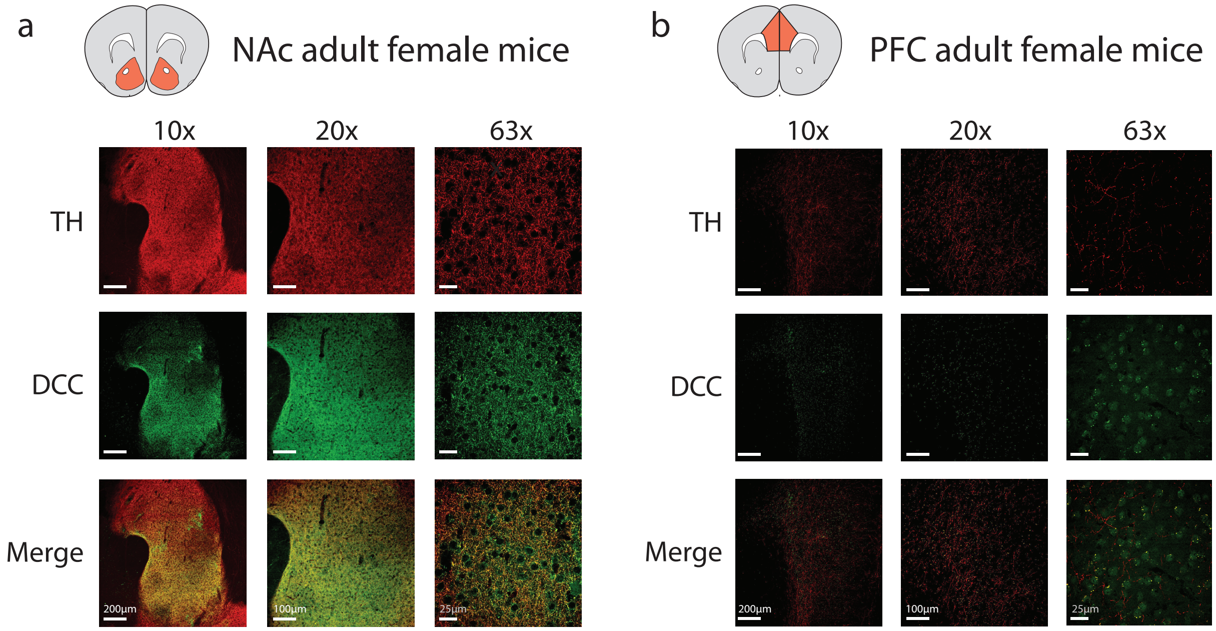

Supplementary figure 1 *DCC protein is highly expressed in mesolimbic, but not mesocortical dopamine axons of adult female mice.* (a) DCC protein is highly expressed in the NAc of adult female mice, with its expression limited to dopamine axons innervating the region. (b) In the PFC local neurons express DCC protein,[1] but it is very seldomly expressed on dopamine axons. This segregation of DCC expression between the mesolimbic and mesocortical dopamine pathways is identical to what has been previously reported in male mice.[2] Representative images are shown, 4 replicates were assessed.

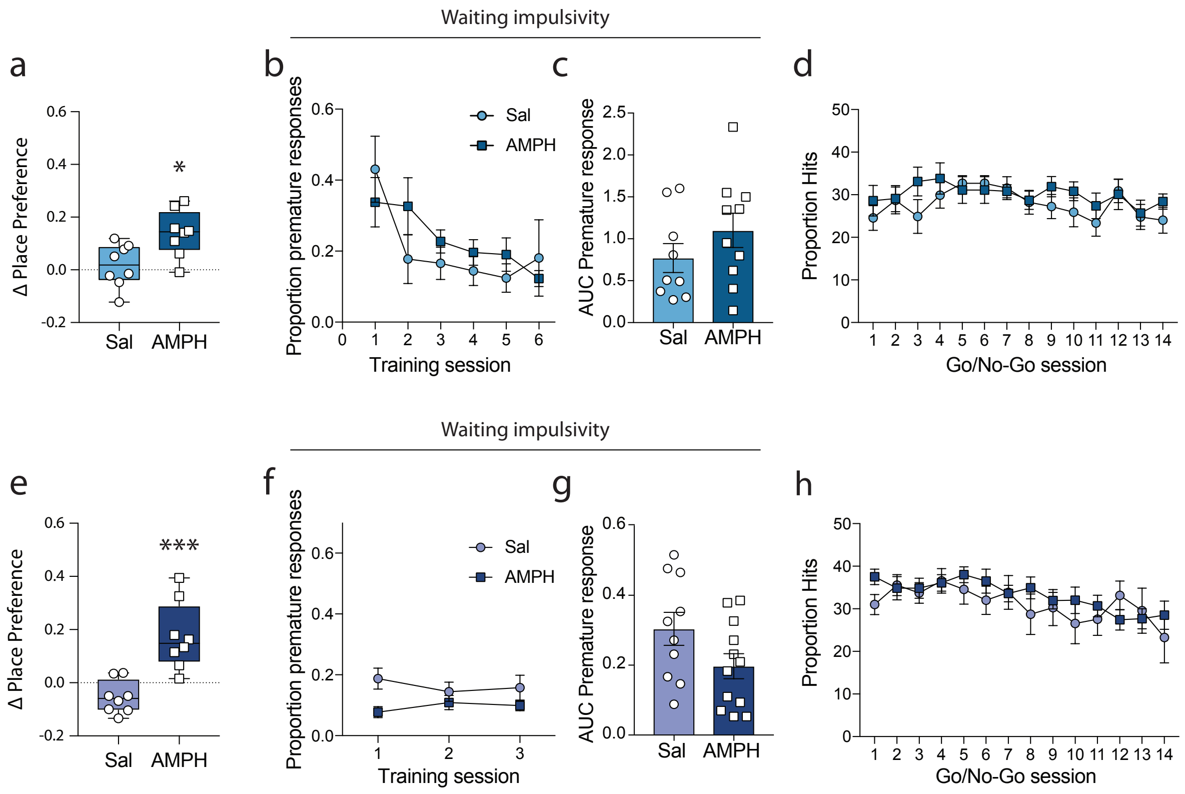

Supplementary figure 2 *Adolescent females are protected from the enduring effects of recreational-like amphetamine.* (a) Female mice show robust place preference to a recreational-like dose of 4 mg/kg amphetamine (AMPH) when tested in early adolescence (P21±1 to P31±1; Supplementary Table 2A). (b-c) Adult female mice treated with AMPH in early adolescence do not show impairments in waiting impulsivity, measured by the level of premature responses during the final training phase of the Go/No-Go task over training days (b) or when assessed by the area under the curve (c) (Supplementary Table 2B,C). (d) Adult female mice treated with AMPH in early adolescence do not show impairments in the correct response to ‘Go’ trials within Go/No-Go task (Supplementary Table 2D). (e) Female mice show robust place preference to AMPH when tested in mid-adolescence (P35±1 to P44±1; Supplementary Table 2E). (f-g) Adult female mice treated with AMPH in mid-adolescence do not show impairments in waiting impulsivity, measured by the level of premature responses during the final training phase of the Go/No-Go task over training days (f) or when assessed by the area under the curve (g) (Supplementary Table 2F,G). (h) Adult female mice treated with AMPH in mid-adolescence do not show impairments in the correct response to ‘Go’ trials within Go/No-Go task (Supplementary Table 2H). All bar and line graphs are presented as mean values±SEM. Source data are provided as a Source Data file. * = p <0.05, *** = p < 0.01.

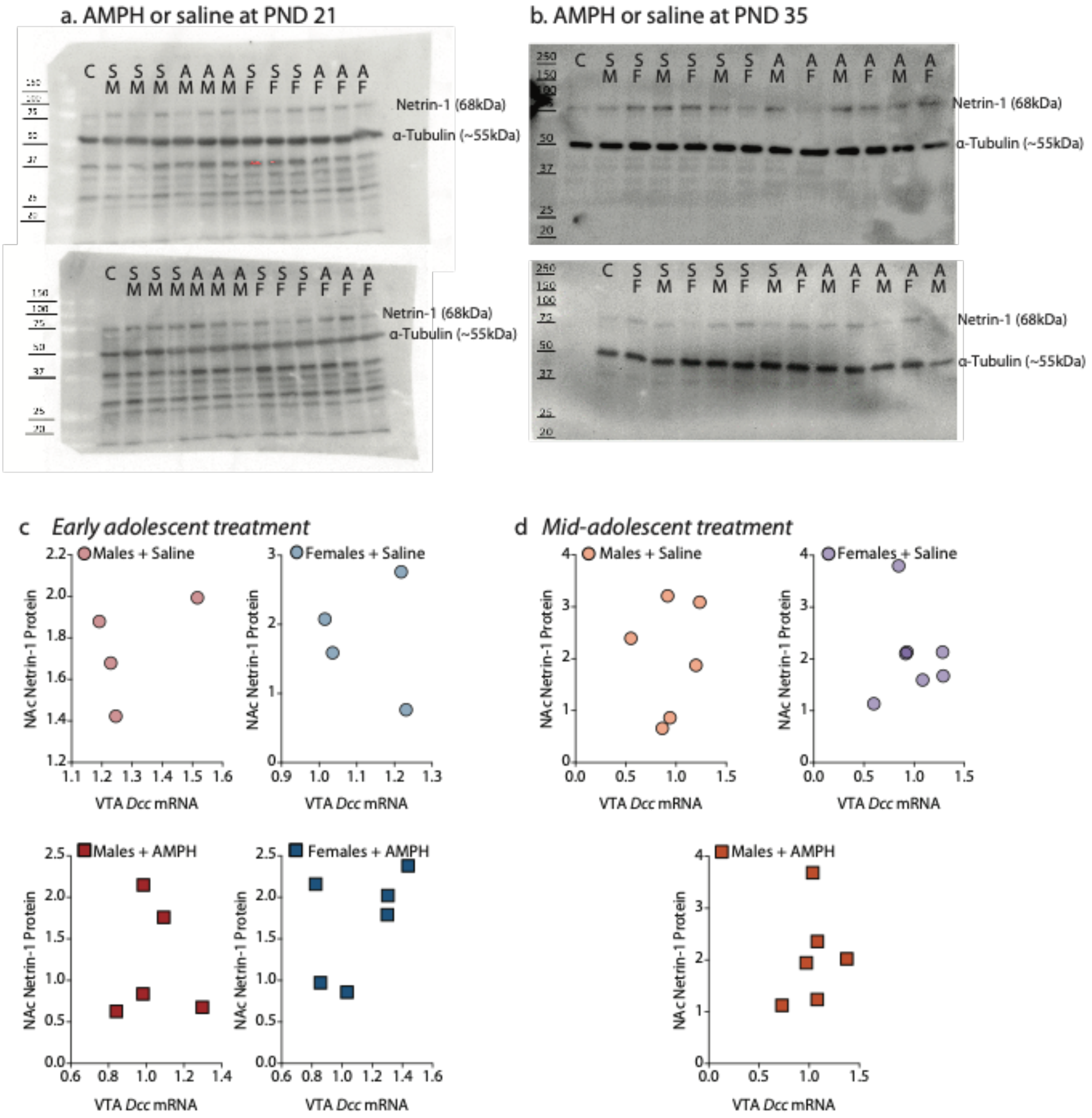

Supplementary figure 3 *Western blots for netrin-1 in the NAc.* Full blots for quantifications shown in Figure 3. (a) Western blot films from male (M) and female (F) mice treated with saline (S) or AMPH (A) in early adolescence (P21±1 to P31±1). (b) Western blot films from male (M) and female (F) mice treated with saline (S) or AMPH (A) in mid-adolescence (P35±1 to P44±1). Netrin-1 bands (68kDa) and α-tubulin reference bands (~55kDa) are indicated for each blot. (c) There was no correlation between Netrin-1 protein in the NAc and *Dcc* mRNA in the VTA for mice treated with AMPH or Saline in early adolescence, regardless of sex (Supplementary Table 3 A). (d) There was no correlation between Netrin-1 protein in the NAc and *Dcc* mRNA in the VTA for mid-adolescent males or female mice treated with saline, nor in mid-adolescent males treated with AMPH (Supplementary Table 3 B).

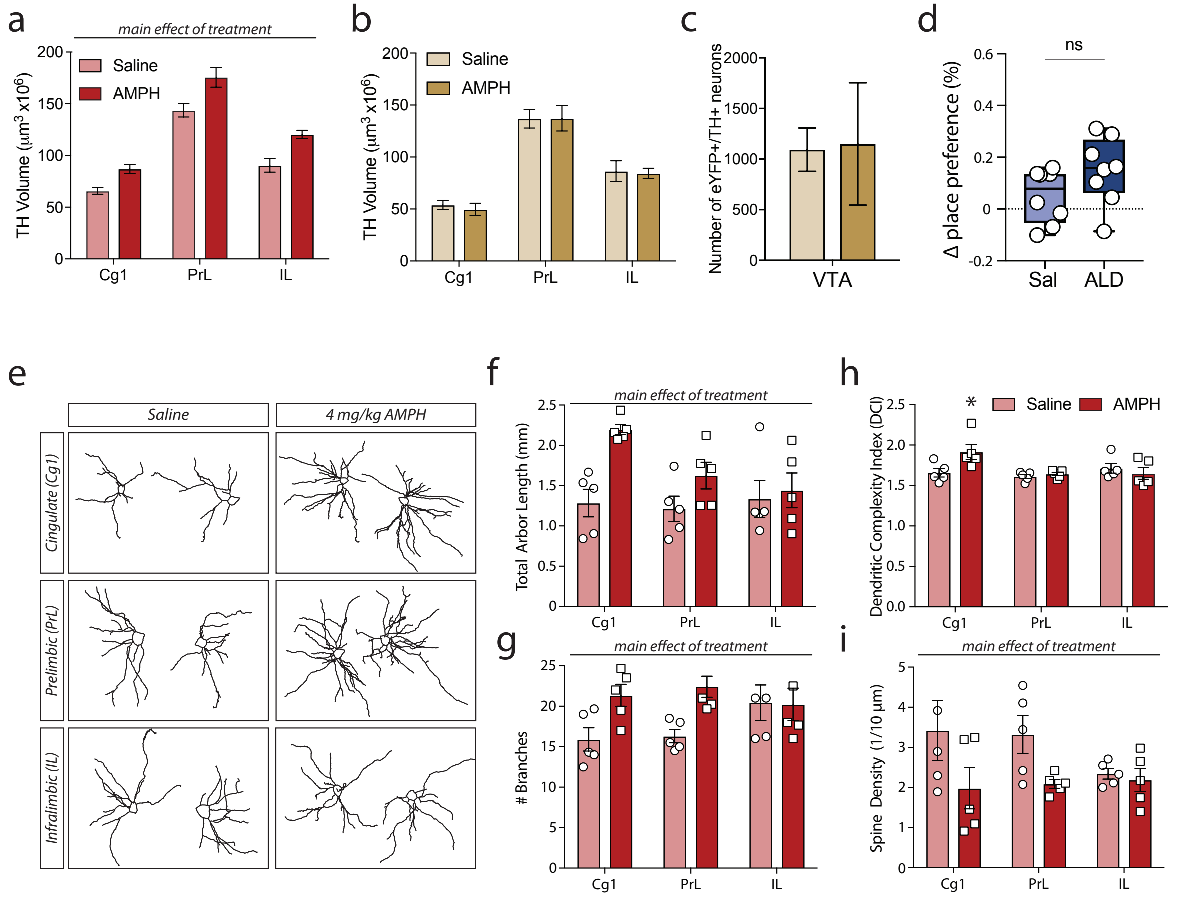

Supplementary figure 4 *Recreational-like AMPH increases dopamine innervation to the PFC and induces enduring changes in pyramidal neuron structure in male mice.* (a) Adult PFC dopamine input volume, measured by TH+ staining to the inner layers of three subregions of the medial PFC (Cg1, PrL, IL) is increased following exposure to recreational-like AMPH in early adolescent (P21±1 to P31±1) male mice (Supplementary Table 4A). (b) Exposure to the therapeutic-like ALD in early adolescent (P21±1 to P31±1) mice does not produce enduring alterations in mPFC dopamine innervation volume (Supplementary Table 4B). (c) Infection levels of VTA dopamine neurons did not differ between saline and ALD-treated mice (Supplementary Table 4C). (d) ALD does not produce place conditioning in mid-adolescent female mice (Supplementary Table 4D). (e) Examples of Neurolucida tracings of PFC golgi-impregnated pyramidal neurons in adult male mice following treatment with saline or AMPH in early adolescence. (f-i) Neurolucida quantification of mPFC pyramidal neuron arbors in adult male mice treated with AMPH or saline in early adolescence. AMPH-treated mice show increases in total arbor length (f, Supplementary Table 4E) and number of branches (g, Supplementary Table 4F) across the three subregions of the mPFC studied. An increase in the complexity of pyramidal neuron arbors was evident only in the Cg1 subregion (h, Supplementary Table 4G), while spine density assessed on tertiary branches was decreased across the PFC (i, Supplementary Table 4H). All

bar and line graphs are presented as mean values±SEM. Source data are provided as a Source Data file. * = $p < 0.05$.

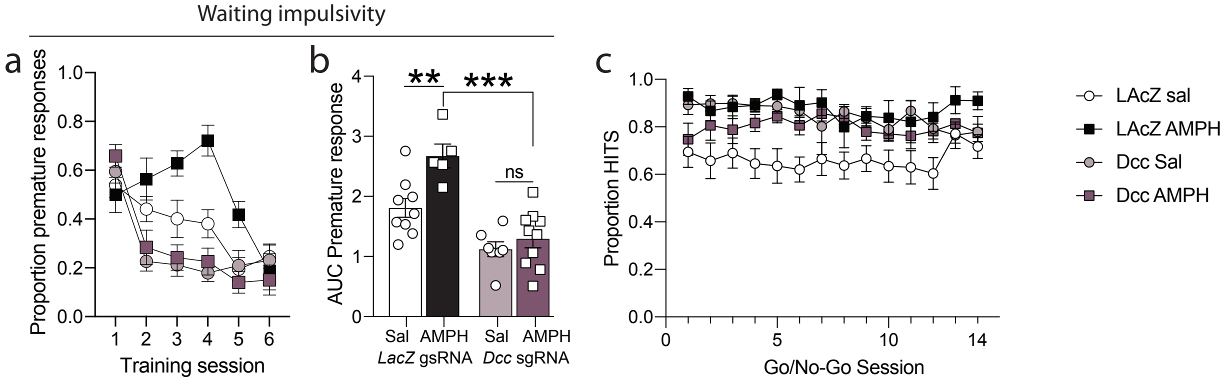

Supplementary figure 5 *CRISPRa upregulation of Dcc expression in the VTA prevents AMPH-induced deficits in waiting impulsivity.* (a-b). Adult male mice that received the *LacZ* sgRNA and were treated with AMPH in early adolescence showed impairments in waiting impulsivity, as measured by a high level of premature responses made over training days during the final training phase of the Go/No-Go task. In contrast, mice that received the *LacZ* sgRNA and were treated with saline and with mice that received the *Dcc* sgRNA and were treated with AMPH or with saline show significant improvement starting on the second training session (a, Supplementary Table 5A). Assessment of the area under the curve (AUC, b, Supplementary Table 5B), revealed that LacZ-injected mice show a significant difference in AUC between Saline and AMPH treated mice, but that this difference is abolished in the mice treated with the *Dcc* sgRNA. (c) No drug x construct x session effect is evident in the proportion of correct response to 'Go' trials (Hits) within the Go/No-Go task (Supplementary Table 5C).All bar and line graphs are presented as mean values±SEM. Source data are provided as a Source Data file. ** = p < 0.01, *** = p < 0.01.

Supplementary Table 1 : CRISPR RNA (crRNA) sequences for constructing single guide RNAs (sgRNAs)				
	**Starting base pair**	**Target sequence**	**sgRNA sense**	**sgRNA anti-sense**
**crRNA 1**	chr18:72351162	GGCCTAGCGAAGCTTAGGGGGGG	CACCGGCCTAGCGAAGCTTAGGGG	AAACCCCCTAAGCTTCGCTAGGCC
**crRNA 2**	chr18:72351390	GGTGTTTCACATAGGGCAAGTGG	CACCGGTGTTTCACATAGGGCAAG	AAACCTTGCCCTATGTGAAACACC
**crRNA 3**	chr18:72351532	CTCGCGTTTGTTTTCCGTGTGGG	CACCCTCGCGTTTGTTTTCCGTG	AAACCACGGAAAACAAACGCGAGC
**crRNA 4**	chr18:72351809	GTTAACACACTCTCATCATACGG	CACCGTTAACACACTCTCATCATA	AAACTATGATGAGAGTGTGTTAAC

Supplementary Table 2: Detailed statistics for Supplementary Figure 2

	Statistical test	Factor	n	Statistic	95% confidence interval	p value (adjusted where appropriate)	corresponding figure
A			8/group				Supplementary Figure 2a
	Two-tailed unpaired t test	Saline vs AMPH CPP		t(14) =2.868	0.03082 to 0.2136	**0.0124**	
B			Saline = 5 AMPH = 6				Supplementary Figure 2b
	Two-way mixed ANOVA	Interaction		F (5, 45) = 0.7547		0.5871	
		Training session (within subject)		F (5, 45) = 4.586		**0.0018**	
		Treatment (between subjects)		F (1, 9) = 0.3616		0.5625	
C			Saline = 5 AMPH = 6				Supplementary Figure 2c
	Two-tailed unpaired t test	AUC		t(17) = 1.219	-0.2409 to 0.8999	0.2396	
D			Saline = 9 AMPH = 10				Supplementary Figure 2d
	Two-way mixed ANOVA	Interaction		F (13, 221) = 0.7133		0.7493	

		Go/No-Go session (within subject)		F (13, 221) = 1.782		**0.0471**		
		Treatment (between subjects)		F (1, 17) = 0.9632		0.3401		
E			8/group				Supplementary Figure 2e	
	Two-tailed unpaired t test	Saline vs AMPH CPP		t(14) =4.55	0.1206 to 0.3357	**0.0005**		
F			Saline = 8 AMPH = 12				Supplementary Figure 2f	
	Two-way mixed ANOVA	Interaction		F (2, 36) = 2.067		0.1413		
		Training session (within subject)		F (2, 36) = 0.04828		0.9529		
		Treatment (between subjects)		F (1, 18) = 4.76		**0.0426**		
G			Saline = 8 AMPH = 12				Supplementary Figure 2g	
	Two-tailed unpaired t test	AUC		t(20)=1.842	-0.2282 to 0.01416	0.0803		
H			Saline = 7 AMPH = 12				Supplementary Figure 2h	
	Two-way mixed ANOVA	Interaction		F (13, 221) = 1.408		0.1569		
		Go/No-Go session (within subject)		F (13, 221) = 4.575		**<0.0001**		

		Treatment (between subjects)		$F (1, 17) = 0.3712$	0.5504		
							**Significant p values are noted in bold text**

Supplementary Table 3: Detailed statistics for Supplementary Figure 3

	Statistical test	Factor	n	Statistic	95% confidence interval	p value (adjusted where appropriate)	corresponding figure	
A							Supplementary Figure 3c	
	Pearson r	Males + saline	4	r = 0.5482, r2 = 0.3005	-0.8727 to 0.9885	0.4518		
		Females + saline	4	r = -0.1112, r2 = 0.01236	-0.9688 to 0.9516	0.8888		
		Males + AMPH	5	r = -0.04895, r2 = 0.002396	-0.8927 to 0.8709	0.9377		
		Females + AMPH	6	r = 0.5152, r2 = 0.2654	-0.5094 to 0.9356	0.2956		
B							Supplementary Figure 3d	
	Pearson r	Males + saline	7	r = -0.02616, r2 =0.0006842	-0.7642 to 0.7415	0.9556		
		Females + saline	6	r = 0.154, r2 = 0.02373	-0.7515 to 0.8583	0.7708		
		Males + AMPH	6	r = 0.2672, r2 = 0.07138	-0.6951 to 0.8865	0.6088		

Supplementary Table 4: Detailed statistics for Supplementary Figure 4

	Statistical test	Factor	n	Statistic	95% confidence interval	p value (adjusted where appropriate)	corresponding figure
**A**			Saline = 5<br>AMPH = 4				Supplementary Figure 4a
	Two-way mixed ANOVA	Interaction		F (2, 14) = 1.002		0.3921	
		Subregion (within subject)		F (2, 14) = 221.9		**<0.0001**	
		Treatment (between subjects)		F (1, 7) = 15.07		**0.006**	
**B**			Saline = 4<br>AMPH = 6				Supplementary Figure 4b
	Two-way mixed ANOVA	Interaction		F (2, 16) = 0.07807		0.9252	
		Subregion (within subject)		F (2, 16) = 111.2		**<0.0001**	
		Treatment (between subjects)		F (1, 8) = 0.03607		0.8541	
C			Saline = 3<br>AMPH = 5				Supplementary Figure 4c

	Two-tailed unpaired t test	Saline vs ALD		t(6)=0.06831	-1954 to 2066	0.9478	
D			8/ group				Supplementary Figure 4d
	Two-tailed unpaired t test	Saline vs AMPH CPP		t(14) =1.676	-2.754 to 22.45	0.1159	
E			5/ group				Supplementary Figure 4d
	Two-way mixed ANOVA	Interaction		F (2, 16) = 3.008		0.0778	
		Subregion (within subject)		F (2, 16) = 2.754		0.0938	
		Treatment (between subjects)		F (1, 8) = 9.272		**0.0159**	
F			5/ group				Supplementary Figure 4g
	Two-way mixed ANOVA	Interaction		F (2, 16) = 3.255		0.0651	
		Subregion (within subject)		F (2, 16) = 0.8069		0.4636	
		Treatment (between subjects)		F (1, 8) = 5.544		**0.0463**	

G			5/ group				Supplementary Figure 4h
	Two-way mixed ANOVA	Interaction		F (2, 16) = 4.064		**0.0374**	
		Subregion (within subject)		F (2, 16) = 3.904		**0.0416**	
		Treatment (between subjects)		F (1, 8) = 2.245		0.1724	
	Sidak's multiple comparisons test	Saline vs. AMPH within Cg1		t(24) = 3.09	0.04403 to 0.475	**0.0149**	
	Sidak's multiple comparisons test	Saline vs. AMPH within PrL		t(24) = 0.3517	-0.186 to 0.245	0.9799	
	Sidak's multiple comparisons test	Saline vs. AMPH within IL		t(24) = 0.7362	-0.2773 to 0.1537	0.85	
H			5/ group				Supplementary Figure 4i
	Two-way mixed ANOVA	Interaction		F (2, 16) = 1.226		0.3195	
		Subregion (within subject)		F (2, 16) = 0.6534		0.5336	
		Treatment (between subjects)		F (1, 8) = 6.85		**0.0308**	
Significant p values are noted in bold text							

Supplementary Table 5: Detailed statistics for Supplementary Figure 5

	Statistical test	Factor	n	Statistic	**95% confidence interval**	**p value (adjusted where appropriate)**	corresponding figure
A			*LacZ* + Saline= 9<br>LacZ + AMPH= 5<br>Dcc + Saline = 8<br>*Dcc* + AMPH = 10				Supplementary Figure 5a
	Generalized Estimating Equations analysis (GEE)	Drug (between subjects)		Wald Chi-Square = 5.408 (df = 1)		**0.02**	
		Construct (between subjects)		Wald Chi-Square = 25.669 (df = 1)		**<0.001**	
		Session (within subjects)		Wald Chi-Square = 127.826 (df = 5)		**<0.001**	
		Drug x Construct		Wald Chi-Square = 4.484 (df = 1)		**0.034**	
		Construct x Session		Wald Chi-Square = 84.410 (df = 5)		**<0.001**	
		Drug x Session		Wald Chi-Square = 27.922 (df = 5)		**<0.001**	
		Drug x Construct x Session		Wald Chi-Square = 22.721 (df = 5)		**<0.001**	
B			*LacZ* + Saline= 9<br>LacZ + AMPH= 5<br>Dcc + Saline = 8				Supplementary Figure 5b

			Dcc + AMPH = 10				
	Two-way ANOVA	Interaction		F (1, 27) = 4.39		**0.0457**	
		sgRNA construct (between subject)		F (1, 27) = 39.45		**<0.0001**	
		Drug (between subject)		F (1, 27) = 9.991		**0.0039**	
	Tukey's multiple comparisons test	LacZ:Sal vs. LacZ:AMPH		q(27) = 4.959	-1.538 to -0.1897	**0.0082**	
	Tukey's multiple comparisons test	LacZ:Sal vs. Dcc:Sal		q(27) = 4.372	**0.0789 to 1.297**	**0.0224**	
	Tukey's multiple comparisons test	LacZ:Sal vs. Dcc:AMPH		q(27) = 3.574	-0.04244 to 1.068	*0.0781*	
	Tukey's multiple comparisons test	LacZ:AMPH vs. Dcc:Sal		q(27) = 8.487	0.8439 to 2.259	**<0.0001**	
	Tukey's multiple comparisons test	LacZ:AMPH vs. Dcc:AMPH		q(27) = 8.049	0.7145 to 2.038	**<0.0001**	
	Tukey's multiple comparisons test	Dcc:Sal vs. Dcc:AMPH		q(27) = 1.138	-0.7705 to 0.4203	0.8516	
C			*LacZ* + Saline= 9<br>LacZ + AMPH= 5<br>Dcc + Saline = 8<br>*Dcc* + AMPH = 10				Supplementary Figure 5c
	Generalized Estimating Equations analysis (GEE)	Drug (between subjects)		Wald Chi-Square = 5.74 (df = 1)		0.17	
		Construct (between subjects)		Wald Chi-Square = 2.292 (df = 1)		0.13	
		Session (within subjects)		Wald Chi-Square = 15.95 (df = 13)		0.252	

		Drug x Construct		Wald Chi-Square = 13.703 (df = 1)	**<0.001**	
		Construct x Session		Wald Chi-Square = 21.309 (df = 13)	0.67	
		Drug x Session		Wald Chi-Square = 62.54 (df = 13)	**<0.001**	
		Drug x Construct x Session		Wald Chi-Square = 17.60 (df = 13)	0.173	
**Significant p values are noted in bold text**						

Supplementary References

1. Torres-Berrío, A. *et al.* DCC Confers Susceptibility to Depression-like Behaviors in Humans and Mice and Is Regulated by miR-218. *Biol Psychiat* 81, 306 315 (2017).

2. Manitt, C. *et al.* The netrin receptor DCC is required in the pubertal organization of mesocortical dopamine circuitry. *J Neurosci* 31, 8381 8394 (2011).
