# Supplementary material for: Amphetamine disrupts dopamine axon growth in adolescence by a sex-specific mechanism in mice
Source: Nat Commun. 2023 Jul 7;14:4035. doi: 10.1038/s41467-023-39665-1 (PMC10329029; doi:10.1038/s41467-023-39665-1)
Supplement: Supplementary file 3 — Reporting Summary [file 41467_2023_39665_MOESM3_ESM.pdf]

nature portfolio

Corresponding author(s): Cecilia Flores

Last updated by author(s): 06/15/2023

Reporting Summary

Nature Portfolio wishes to improve the reproducibility of the work that we publish. This form provides structure for consistency and transparency in reporting. For further information on Nature Portfolio policies, see our Editorial Policies and the Editorial Policy Checklist.

Statistics

For all statistical analyses, confirm that the following items are present in the figure legend, table legend, main text, or Methods section.

n/a	Confirmed	
☐	☒	The exact sample size (*n*) for each experimental group/condition, given as a discrete number and unit of measurement
☐	☒	A statement on whether measurements were taken from distinct samples or whether the same sample was measured repeatedly
☐	☒	The statistical test(s) used AND whether they are one- or two-sided<br>*Only common tests should be described solely by name; describe more complex techniques in the Methods section.*
☒	☐	A description of all covariates tested
☐	☒	A description of any assumptions or corrections, such as tests of normality and adjustment for multiple comparisons
☐	☒	A full description of the statistical parameters including central tendency (e.g. means) or other basic estimates (e.g. regression coefficient) AND variation (e.g. standard deviation) or associated estimates of uncertainty (e.g. confidence intervals)
☐	☒	For null hypothesis testing, the test statistic (e.g. *F*, *t*, *r*) with confidence intervals, effect sizes, degrees of freedom and *P* value noted<br>*Give P values as exact values whenever suitable.*
☒	☐	For Bayesian analysis, information on the choice of priors and Markov chain Monte Carlo settings
☒	☐	For hierarchical and complex designs, identification of the appropriate level for tests and full reporting of outcomes
☐	☒	Estimates of effect sizes (e.g. Cohen's *d*, Pearson's *r*), indicating how they were calculated

Our web collection on statistics for biologists contains articles on many of the points above.

Software and code

Policy information about availability of computer code

Data collection	Stereoinvestigator 2021.1.1, Neurolucida 2022.1.1, MedPC IV, BioRad Image Lab 6.1
Data analysis	GraphPad Prism 9.4.0, SPSS 28.0.0.0

For manuscripts utilizing custom algorithms or software that are central to the research but not yet described in published literature, software must be made available to editors and reviewers. We strongly encourage code deposition in a community repository (e.g. GitHub). See the Nature Portfolio guidelines for submitting code & software for further information.

Data

Policy information about availability of data

All manuscripts must include a data availability statement. This statement should provide the following information, where applicable:

- Accession codes, unique identifiers, or web links for publicly available datasets
- A description of any restrictions on data availability
- For clinical datasets or third party data, please ensure that the statement adheres to our policy

Uncropped blot images are available in Extended Data Figure 3. All other source data are provided with this paper.

Human research participants

Policy information about studies involving human research participants and Sex and Gender in Research.

Reporting on sex and gender	n/a
Population characteristics	n/a
Recruitment	n/a
Ethics oversight	n/a

Note that full information on the approval of the study protocol must also be provided in the manuscript.

Field-specific reporting

Please select the one below that is the best fit for your research. If you are not sure, read the appropriate sections before making your selection.

☒ Life sciences ☐ Behavioural & social sciences ☐ Ecological, evolutionary & environmental sciences

For a reference copy of the document with all sections, see nature.com/documents/nr-reporting-summary-flat.pdf

Life sciences study design

All studies must disclose on these points even when the disclosure is negative.

Sample size	No sample size calculations were performed. Suitable sample sizes were estimated based on previous experiments (e.g. Reynolds et al 2018) and are similar to those employed in the field.
Data exclusions	Mice that did not learn the No-Go contingency of the Go/No-Go task were identified by a low rate of correct responses to the Go contingency (< 50%) and excluded. We did not exclude animals from any experiment using statistical outliers.
Replication	All experiments were replicated with success, meaning that group measures (mean, SD) did not vary significantly across experimental cohorts (minimum of 2) tested on separate occasions.
Randomization	Mice were pseudorandomly assigned to experimental groups, with counterbalancing in consideration for minimizing litter/cagemate effects. For all other experiments samples were fully randomized.
Blinding	Experimenters were blinded to group allocation during data collection and analysis.

Reporting for specific materials, systems and methods

We require information from authors about some types of materials, experimental systems and methods used in many studies. Here, indicate whether each material, system or method listed is relevant to your study. If you are not sure if a list item applies to your research, read the appropriate section before selecting a response.

Materials & experimental systems

n/a	Involved in the study	
☐	☒	Antibodies
☐	☒	Eukaryotic cell lines
☒	☐	Palaeontology and archaeology
☐	☒	Animals and other organisms
☒	☐	Clinical data
☒	☐	Dual use research of concern

Methods

n/a	Involved in the study	
☒	☐	ChIP-seq
☒	☐	Flow cytometry
☒	☐	MRI-based neuroimaging

Antibodies

Antibodies used	polyclonal rabbit anti-TH antibody (1:1000, cat. no. AB152, Millipore Sigma, USA); polyclonal chicken anti-TH (Aves lab, AB_10013440); monoclonal mouse anti-TH antibody (Millipore Sigma, cat. no. MAB318); polyclonal anti-GFP raised in chicken (1:1000, antibody #1020, Aves labs); polyclonal rabbit anti-RFP (Rockland, cat. no. 600-401-379); polyclonal rabbit anti-DCC (antibody #2473, Dr. H. M. Cooper, University of Queensland, Brisbane, QLD, Australia)

	anti-mouse Alexa Fluor-488 conjugated secondary antibody , (1:1000, Invitrogen cat. no. A-11001) ; anti-chicken Alexa Fluor 488-conjugated secondary antibody raised in goat (1:500; Invitrogen cat. no. A11039); donkey anti-rabbit Alexa Fluor 594 (Invitrogen, cat. no. A-21207)
Validation	polyclonal rabbit anti-TH antibody AB152<br>https://www.merckmillipore.com/FR/fr/product/Anti-Tyrosine-Hydroxylase-Antibody,MM_NF-AB152<br><br>monoclonal mouse anti-TH antibody (Millipore Sigma, cat. no. MAB318)<br>https://www.sigmaaldrich.com/FR/fr/product/mm/mab318<br><br>polyclonal rabbit anti-DCC (antibody #2473, Dr. H. M. Cooper, University of Queensland, Brisbane, QLD, Australia)<br>Seaman et al., 2001 https://doi.org/10.1016/S0925-4773(01)00350-1<br><br>polyclonal chicken anti-GFP<br>https://www.aveslabs.com/products/anti-green-fluorescent-protein-antibody-gfp<br><br>Rockland rabbit anti-RFP<br>https://www.rockland.com/categories/primary-antibodies/rfp-antibody-pre-adsorbed-600-401-379/<br><br>AF conjugated secondaries<br>https://www.thermofisher.com/fr/fr/home/life-science/antibodies/secondary-antibodies/fluorescent-secondary-antibodies/alexa-fluor-secondary-antibodies.html<br><br>All antibodies have, in addition, been previously validated in publications from our lab or our collaborators.

Eukaryotic cell lines

Policy information about cell lines and Sex and Gender in Research

Cell line source(s)	Primary mesencephalic neuron cultures were prepared from dissections of male and female postnatal day 0-2 (P0 to P2) C57/BL6J mice. HEK293T cells from ATCC (catalog number: CRL-3216) were used for lentivirus preparation.
Authentication	no procedure
Mycoplasma contamination	cell lines were not tested for mycoplasma contamination
Commonly misidentified lines<br>(See ICLAC register)	no commonly misidentified cell lines were used in the study

Animals and other research organisms

Policy information about studies involving animals; ARRIVE guidelines recommended for reporting animal research, and Sex and Gender in Research

Laboratory animals	DATCre or wildtype C57BL/6J mice were used starting from PND21
Wild animals	No wild animals were used in the study.
Reporting on sex	Sex was considered in study design and all experiments were performed in both sexes with comparisons between both sexes where applicable.
Field-collected samples	No field collected samples were used in the study
Ethics oversight	Canadian Council of Animal Care and the McGill University/Douglas Mental Health University Institute Animal Care Committee

Note that full information on the approval of the study protocol must also be provided in the manuscript.
